# Genomic insights into *Verticillium*: a review of progress in the genomics era

**DOI:** 10.3389/fmicb.2024.1463779

**Published:** 2024-10-11

**Authors:** M. Sayari, A. Dolatabadian, M. El-Shetehy, F. Daayf

**Affiliations:** ^1^Department of Plant Science, Faculty of Agricultural and Food Sciences, University of Manitoba, Winnipeg, MB, Canada; ^2^School of Biological Sciences, The University of Western Australia, Crawley, WA, Australia; ^3^Department of Botany and Microbiology, Faculty of Science, Tanta University, Tanta, Egypt

**Keywords:** genomics, Verticillium, pathogenesis, plant-pathogen interactions, soil-borne fungi

## Abstract

Genomics has emerged as a great tool in enhancing our understanding of the biology of Verticillium species and their interactions with the host plants. Through different genomic approaches, researchers have gained insights into genes, pathways and virulence factors that play crucial roles in both Verticillium pathogenesis and the defense responses of their host organisms. This review emphasizes the significance of genomics in uncovering the mechanisms that underlie pathogenicity, virulence, and host resistance in Verticillium fungi. Our goal is to summarize recent discoveries in Verticillium research highlighting progress made in comprehending the biology and interactions of Verticillium fungi. The integration of genomics into Verticillium studies has the potential to open avenues for developing strategies to control diseases and produce crop varieties resistant to verticillium, thereby offering sustainable solutions for enhancing agricultural productivity.

## 1 Introduction

The Verticillium genus consists of a group of sac fungi that reproduce both sexually and asexually. These fungi, primarily found in soil, are known for causing damage to crops worldwide and result in substantial economic losses (Inderbitzin et al., 2011). The genus was first described by Friedrich Traugott Cutzing in 1816, who noted the whorled arrangement of their conidiophores. Since then, researchers have identified more than 190 species within this group, with *Verticillium dahliae* and *V. alboatrum* being the most extensively studied and economically important (Inderbitzin et al., 2011).

The taxonomic classification of Verticillium has been a topic of debate leading to multiple rounds of revisions over time (Zare et al., 2007). In 2011, a comprehensive taxonomic revision resulted in the establishment of a genus called Verticillium *sensu stricto*. This new classification included 10 species; *Verticillium dahliae*, *V. alfalfae*, *V. nubilum*, *V. isaacii*, *V. klebahnii*, *V. zaregamsianum*, *V. longisporum*, *V. nonalfalfae*, *V. tricorpus*, and *V. albo-atrum* (Inderbitzin et al., 2011). Through advanced molecular analysis, *V. theobromae* and *V. nigrescens* were reclassified and relocated to other genera (Zare et al., 2007). Future advances may add other species to this group.

**Figure 1:**
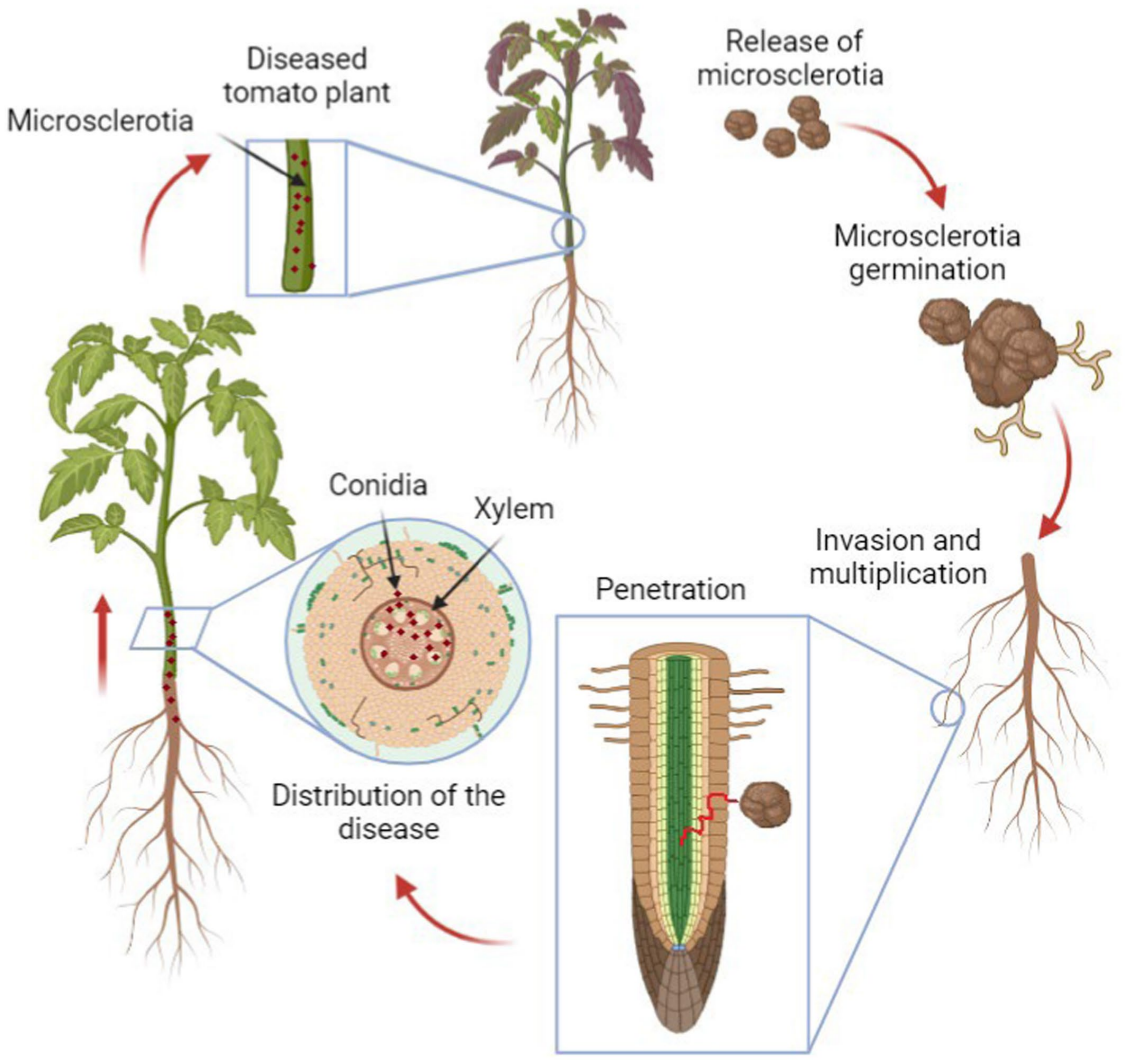
The life cycle of *Verticillium* in plants. The cycle begins with fungal spores (microsclerotia) in the soil, which germinate and infect the plant roots. The fungus then spreads through the vascular system, causing wilting and other symptoms. Eventually, the fungus produces new microsclerotia within the dying plant tissue, which return to the soil, ready to infect new hosts.

The biology of Verticillium is complex and still not completely understood, as the fungus goes through both its saprophytic phase where it breaks down dead organic matter in the soil and a parasitic phase where it infects host plants (López-Escudero and Mercado-Blanco, 2011). The infection process (Figure 1) starts with the hyphae penetrating the roots of host plants, colonizing the xylem ducts and then causing systemic infection that eventually leads to wilting and death of the entire plant (López-Escudero and Mercado-Blanco, 2011).

**Figure 2:**
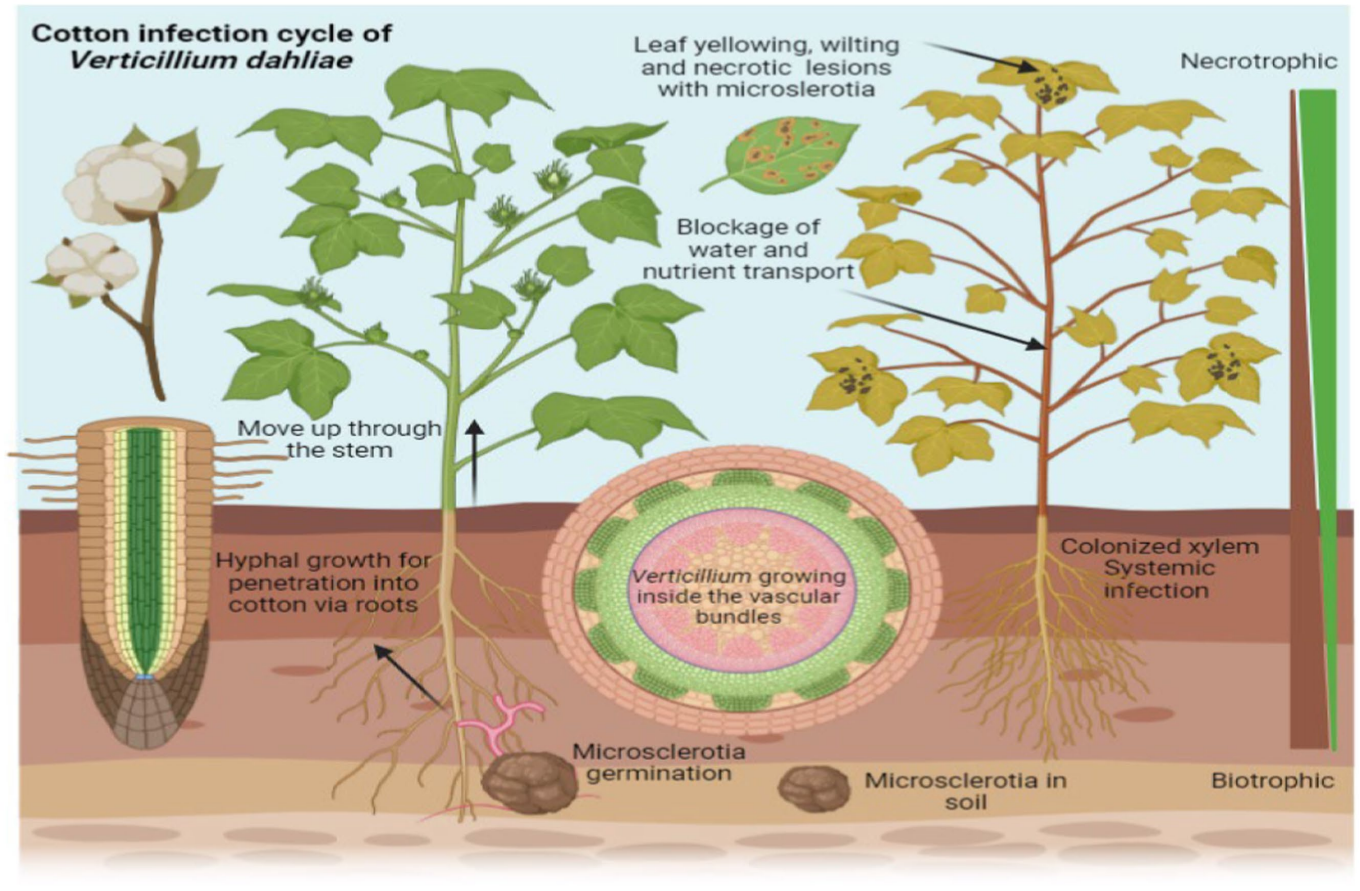
*Verticillium* wilt in cotton plants caused by the fungus *Verticillium dahliae*. The image illustrates the progression of the disease, showing wilting and yellowing of leaves as well as stunted growth. These symptoms result from the pathogen’s invasion of the plant’s vascular system, which blocks the transport of water and nutrients, ultimately leading to a reduction in yield.

Verticillium species have a range of hosts, infecting more than 200 plant species, including important crops such as cotton (*Gossypium arboreum* L.), tomato (*Solanum lycopersicum*), potato (*Solanum tuberosum*), and strawberry (*Fragaria ananassa*) (Klosterman et al., 2011). Verticillium wilt is a significant disease affecting cotton plants, caused by the soil-borne fungus *V. dahliae*. This pathogen invades the plant’s vascular system, leading to the blockage of water and nutrient transport. As the infection progresses, cotton plants exhibit symptoms such as wilting, yellowing of leaves, and stunted growth, which ultimately reduces yield (Figure 2). The virulence of Verticillium is mainly attributed to its wilting effects and production of toxins, both resulting in leaf and stem yellowing and necrosis. These symptoms can result in crop losses reaching up to 80% (Fradin and Thomma, 2006), thereby inflicting terrible impact on farmers and the agricultural industry (López-Escudero and Mercado-Blanco, 2011). There have been various reports of crop damage caused by verticillium wilt, in many different countries, including but not limited to the United States, China, Canada, and Tunisia (Daayf, 2015; Gharbi et al., 2015). Notably, Canada experiences losses of millions of dollars in potato crops due to this disease (Molina et al., 2014).

Over time, researchers have shown great interest in studying Verticillium species. This has led to investigating their diversity, pathogenicity, and management strategies (Song et al., 2020; Zhang et al., 2022; Barbara and Clewes, 2003; Luo et al., 2014). Advanced reviews (Klosterman et al., 2009; Daayf, 2015) provide insights into different aspects of Verticillium species such as their host ranges, mechanisms of pathogenicity as well as management strategies. Subsequent studies have delved into the molecular mechanisms behind plant resistance to Verticillium (Song et al., 2020) as well as the challenges involved in distinguishing phytopathogenic from non-pathogenic Verticillium strains (Barbara and Clewes, 2003). Several studies have provided insights into the ways *V. dahliae* causes disease in host plants (Luo et al., 2014; Zhang et al., 2022). Carroll et al. (2018) extensively examined strategies and unintended consequences, for controlling verticillium wilt. Together, previous studies emphasized the importance of understanding the mechanisms behind Verticillium pathogenesis and the intricate nature of its system to effectively manage verticillium wilt disease in the genomics era.

The study of genomics has brought about a transformation in Verticillium research. In 2011, the first Verticillium genome (*V. dahliae*) was sequenced using a combination of Sanger sequencing and 454 pyrosequencing technologies (Klosterman et al., 2011). Since then, several other genomes of Verticillium species such as *V. albo atrum*, *V. nonalfalfae*, and *V. longisporum* have been sequenced (Faino et al., 2016; Depotter et al., 2019). So far, a total of 10 Verticillium species’ genomes have been deposited in the NCBI database (Table 1). These publicly available genome sequences have paved the way for functional genomic studies that enhance our understanding of Verticillium biology, evolution, and diversity (de Jonge et al., 2011). Overcoming challenges related to the size and repetitiveness of genomes as well as the lack of resources and tools for studying fungal plant pathogens, novel sequencing technologies like Illumina and PacBio have greatly simplified the process of sequencing and assembling complex fungal genomes (Aragona et al., 2022). Consequently, transcriptomic and proteomic datasets have been generated to gain insights into gene expression and regulation in Verticillium (Zhang et al., 2020; Jin et al., 2017).

**Table 1:** *Verticillium* genomes with completed genome sequences.

Species	Isolate number	Accession number	Size (Mb)	GC%	Reference
*V. dahliae*	VdLs.17	ABJE00000000	34.71	54	Klosterman et al. (2011)
*V. albo-atrum*	PD747	NMXJ00000000	36.82	56.45	Shi-Kunne et al. (2018)
*V. isaacii*	PD618	NMXN00000000	35.69	57.5	Shi-Kunne et al. (2018)
*V.alfalfae*	VaMs.102	ABPE00000000	32.75	55.4	Klosterman et al. (2011)
*V. nonalfalfae*	VnAa140/PSU140/NRRL 66861	RBVV00000000	33.62	55	Kasson et al. (2019)
*V. nubilum*	PD621	NMXI00000000	37.91	53.7	Shi-Kunne et al. (2018)
*V. zaregamsianum*	PD739	NMXM00000000	37.13	57.5	Shi-Kunne et al. (2018)
*V. longisporum*	VL1	JAETXT000000000	72.48	52.9	Harting et al. (2021)
*V. klebahnii*	PD401	NMXL00000000	36.	57.6	Shi-Kunne et al. (2018)
*V. tricorpus*	MUCL 9792	JPET00000000	35.59	57.4	Seidl et al. (2015)

Researchers have made good progress in understanding Verticillium genomics, thus leading to the discovery of signaling pathways genes and potential targets for controlling diseases caused by the members of this genus (Klimes et al., 2015). Moreover, genomics has played a significant role in developing markers to enhance resistance against Verticillium and create resilient crop products (Zhao et al., 2021; Inderbitzin et al., 2019). The study of Verticillium genomics has also expedited the identification of virulence factors for pathogenesis. For instance, we had pinpointed isochrismatase hydrolase and cupin domain containing proteins with quercetinase activity as important pathogenicity factors of *V. dahliae* (El Hadrami et al., 2015; Zhu et al., 2017). Moreover, by utilizing genomics techniques, we successfully created markers to investigate the diversity within different Verticillium isolates (Gharbi et al., 2015), and more recently, a genome-based analysis focused on the Verticillium polyketide synthase (PKS) gene cluster for synthesizing polyketides which are essential for *V. dahliae*’s virulence (Sayari et al., 2022). Other studies have explored the significance of NADPH oxidase A (NOX A) protein in facilitating the penetration and virulence capabilities of *V. dahliae* (Zhu et al., 2021a).

Comparative studies of genomes have identified genes that are conserved among Verticillium species. These genes, such as secreted proteins, and carbohydrate-activating enzymes (CAZymes) potentially play a crucial role in the virulence of Verticillium species (Wang et al., 2021; Leonard et al., 2020). Through proteomic and transcriptomics, scientists have examined how these genes are expressed during infection, which has led to the discovery of factors contributing to virulence (Chen et al., 2023; Leonard et al., 2020; Wang et al., 2023). The findings from the above-mentioned studies offer new opportunities, for developing strategies to control the diseases caused by Verticillium species, including the development of new fungicides.

**Figure 3:**
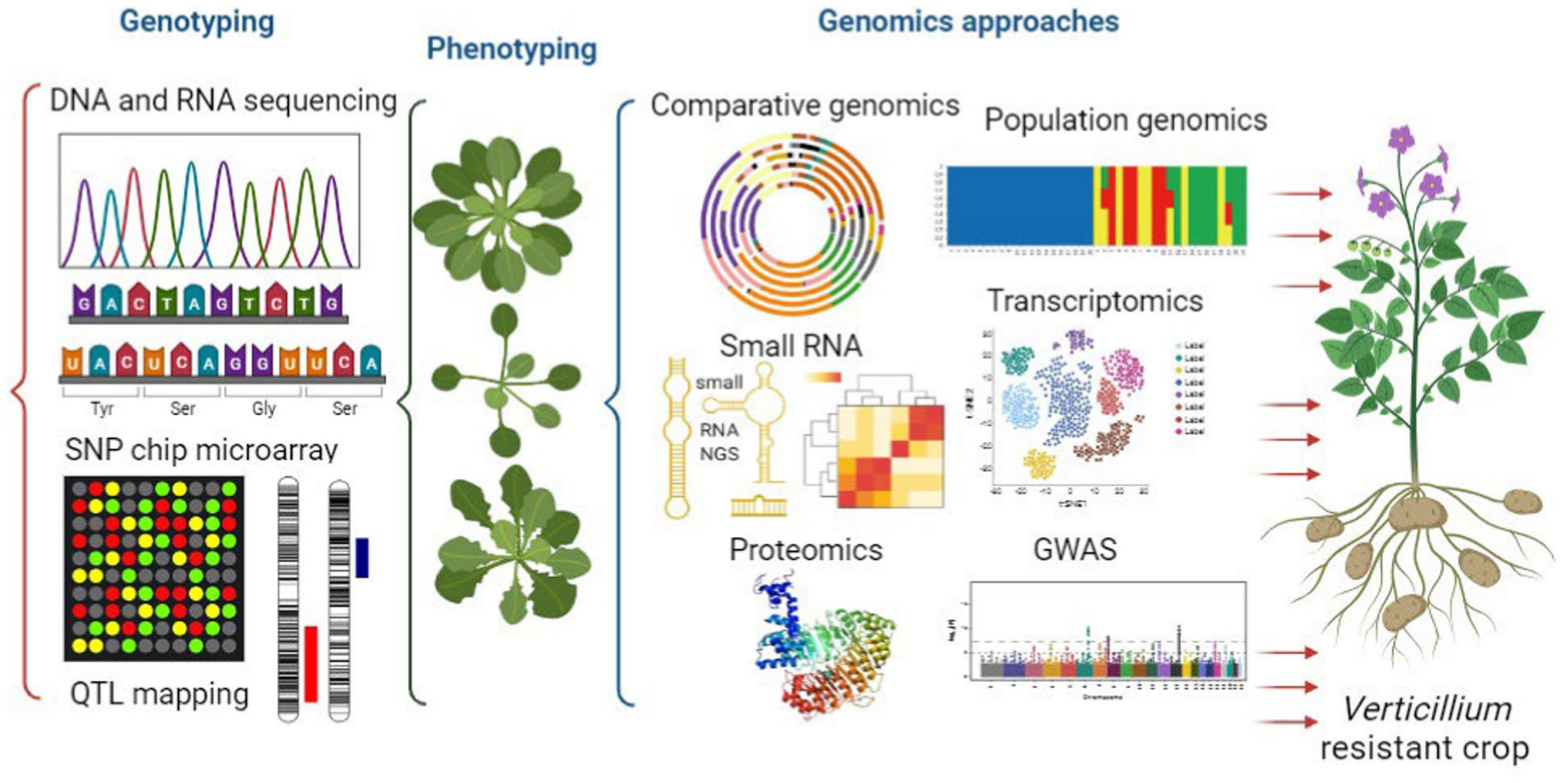
Integrated genomics unraveling *verticillium* pathogenesis toward the development of *verticillium*-resistant crops. This figure illustrates the powerful integration of genomics approaches in unraveling the complex pathogenesis of *Verticillium*. Genomics techniques, including comparative genomics and population genomics, investigation of small RNA and the application of functional genomics (transcriptomics, proteomics, and genome-wide association studies), collectively shed light on the intricate mechanisms underlying *Verticillium*-induced crop diseases.

This review aims to offer an overview of the current state of Verticillium genomics (Figure 3). It focuses on different aspects such as genomics, population genomics and horizontal gene transfer. These genomic approaches can help us understand the evolution and diversity of the members of this genus. Additionally, the review explores the role of RNAs and epigenetic regulation in Verticillium and how they influence gene expression and pathogenicity. The paper also discusses how genomics approaches, and complex networks are used to study crop diseases caused by Verticillium. Moreover, it covers functional genomics techniques such as transcriptomics, proteomics, transformation, as well as genome wide association studies. These methods provide valuable insights into Verticillium’s pathogenicity as well as host resistance. Overall, this review emphasizes the importance of genomics in advancing our knowledge of Verticillium biology, pathogenesis, and the potential for protecting crops from Verticillium-related diseases. Finally, it addresses the challenges and opportunities in genomics research related to crop protection and disease management.

## 2 Genomic analysis and evolutionary dynamics

Genomic analysis has provided profound insights into the evolutionary dynamics of Verticillium species, revealing how these pathogens adapt to various environmental pressures and host plants. Through the study of their genomes, researchers have uncovered genetic variations and evolutionary mechanisms that contribute to their pathogenicity and host specificity. Understanding these dynamics is crucial for developing effective strategies to manage the diseases caused by Verticillium species. This section delves into the key aspects of comparative and population genomics in Verticillium, shedding light on the genetic factors driving their evolution and diversification.

### 2.1 Comparative genomics

Comparative genomics plays a role in the study of *Verticillium* species’ evolution, diversity, and pathogenicity. Analyzing the genomes of *Verticillium* species uncover genes and genetic elements essential for pathogenicity, host specificity and adaptation of the pathogen in different environments (Klosterman et al., 2011). A significant breakthrough in genomics occurred when scientists sequenced and annotated the genomes of *Verticillium* species such as *V. dahliae* and *V. albo-atrum*. Through comparative analysis of these genomes, researchers have discovered variations in genome size, gene content and gene expression patterns across these species, indicating distinct lifestyles and preferences for specific hosts (Klosterman et al., 2011). For example, *V. dahliae* has a larger genome compared to *V. albo-atrum,* which is enriched with genes encoding secretory proteins, carbohydrate processing as well as secondary metabolite biosynthesis. Overall, their research laid a robust groundwork for future studies, including functional analyses of polysaccharide lyases and genes located in lineage-specific regions. Comparative genomic studies have also provided insights into *Verticillium* species’ similarities and differences in virulence mechanisms. In-depth investigations have identified virulence factors specific to certain species; one example is the fujikurine biosynthesis gene clusters found exclusively within the genomes of *V. dahliae* and *V. longisporum* (Sayari et al., 2022). Furthermore, Ingram et al. (2020) compared the genomes of 18 tomato isolates of *V. dahliae*, revealing race features that contribute to host specificity and virulence. Their study identified seven effectors in highly variable genome regions, with race 2 resistance in tomatoes controlled by a single dominant locus, highlighting the need for modern genomics in breeding for durable resistance.

In another study, Depotter et al. (2019) investigated the genomic regions of *V. dahliae* that are related to its virulence, and they successfully identified effector genes that exhibit sequence conservation. Gleeson (2015) employed genomics to examine how *V. dahliae* interacts with cover crops and wild strawberries, focusing on host-pathogen interactions. This investigation discovered genes for virulence and host specificity, including those encoding effector proteins and enzymes that degrade cell walls. By utilizing a genome-based approach, Sayari et al. (2022) successfully analyzed PKS gene clusters in all available genomes of *Verticillium* species. Furthermore, in a recent publication, Arseneault et al. (2023) presented genome data from 192 *V. dahliae* isolates that infect potatoes across Canada. Their findings highlighted the diversity among *V. dahliae* strains originating from different regions in Canada. Comparative genomic studies have also revealed shared genes and pathways between *Verticillium* species and other fungal plant pathogens, such as Fusarium, Coletotrichum, and Botrytis, which are involved in producing phytotoxins carbohydrate-active enzymes as well as secreted proteins (Sayari et al., 2022; de Jonge et al., 2011).

In addition, the comparison genomics has shown how horizontal gene transfer influences the virulence of plant pathogens, with evidence that these pathogens acquired genes from other bacterial and fungal species (Shi-Kunne et al., 2019a,b). Comparative genomic research has also played an important role in understanding the relationships between *Verticillium* species and other fungal plant pathogens. For instance, Seidl et al. (2020) investigated repetitive elements and their impact on centromere diversity and evolution within the fungal genus *Verticillium*. This study provided insights into the epigenetic processes of centromere development among these fungi. Another study by Gibriel (2019) focused on the evolution of virulence in two Ascomycete plant pathogens, *Zymoseptoria tritici* and *V. dahlia*, shedding light on the underlying factors contributing to their pathogenicity. In another study, Fogelqvist et al. (2018) performed the genome analysis of *V. longisporum*. Their findings revealed regions of nonparental origin within its hybrid genome, suggesting that interspecific hybridization played a significant role in the evolution of *V. longisporum*.

Researchers have also identified genes and proteins associated with virulence and host specificity in *V. dahliae*, such as VdSsk2 and VdSte11. These genes are crucial for growth, virulence, and stress response (Yu et al., 2019). Comparative genomic studies have uncovered regions linked to hosts’ virulence and resistance. This valuable information assists us to have a much better understanding of the factors that contribute to the nature of these organisms (Harting et al., 2021; Li H. et al., 2020).

These comparative genomic studies have provided insights into the determinants of pathogenicity and identified potential targets for developing new strategies to combat verticillium wilt disease. For instance, Hu et al. (2014) studied the germination process of *V. dahliae* sclerotia using expression profiles, which revealed critical genetic mechanisms underlying this pathogen’s early stages of infection. Shi-Kunne et al. (2019a) employed computational prediction techniques to identify clusters of secondary metabolites in *V. dahliae,* suggesting their role in virulence and disease control. Chen et al. (2023) discovered sugar transporter genes that influence *V. dahliae’s* virulence on cotton plants, while Chavarro-Carrero et al. (2021) uncovered a particular Av2 effector protein recognized by tomato plants with a V2 resistance locus shedding light on plant-pathogen interactions. Effector proteins play crucial roles in determining the virulence of *Verticillium* species, making them an attractive target for developing methods to manage these diseases, such as making resilient cultivars or fungicides. Horizontal gene transfer events from bacterial species have also been suggested to play a role in acquiring effectors (Shi-Kunne et al., 2019a,b). In the coming section, we will discuss the role of horizontal gene transfer in verticillium pathogenicity.

Comparative genomics has revealed the evolutionary journey of *Verticillium* species. Depotter et al. (2021) reconstructed the phylogenetic relationships among 16 *Verticillium* species, uncovering speciation, hybridization and gene exchange. Genomic regions that undergo evolution may contribute to the pathogen adaptation to different hosts and environments. Harting et al. (2021) identified a region found exclusively in virulent *V. longisporum* isolates, which is crucial for their pathogenicity ability. Li X. et al. (2020) reported the sequences of *V. dahliae* strain XJ592 and non-deciduous strain XJ511, providing insights into the factors determining their pathogenicity ability. Zhang et al. (2021) shared genome sequence data for MAT1-1 and MAT1-2 idiomorphs of *V. dahliae*, enhancing our understanding of its evolution and adaptation.

Overall, comparative genomics is a powerful tool for understanding *Verticillium* species, revealing key aspects of their evolution and pathogenicity. This approach allows researchers to pinpoint specific genes and genetic elements linked to pathogenicity, host specificity, and adaptation. Moreover, by uncovering genetic similarities with other fungal pathogens, it broadens our understanding of fungal pathogenicity. As the field progresses, comparative genomics will continue to identify genetic determinants of virulence and adaptation, aiding in the development of effective disease control measures.

### 2.2 Population genomics

Population genomic studies have provided insights into *Verticillium* species’ diversity and population structure. In a study conducted by Zhang et al. (2019), the genome of *V. dahliae* was analyzed, and specific genomic regions associated with the defoliation phenotype were identified, indicating adaptation to host plants. Their research revealed that the defoliation and high virulence of the D pathotype are caused by the secondary metabolite NAE 12:0, whose biosynthesis is governed by genes within the lineage-specific region G-LSR2 in *V. dahliae*. Another study by Chen et al. (2021) focused on the *V. dahliae* race, revealing that host plants and management practices impact this pathogen’s population structure. They observed that different host plants were linked to subpopulations of *V. dahliae,* suggesting that host-specific adaptations play a vital role in the evolution of this important plant pathogen.

In an investigation carried out by Wang et al. (2021), the genetic basis behind the divergence of cultivars in *V. dahliae* was explored using comparative genomics, transcriptomics, and analysis of lineage-specific regions. Their findings indicated that cultivar divergence in *V. dahliae* was associated with acquiring effector genes important for interactions between hosts and pathogens. Similarly, Wheeler et al. (2019) examined endophyte populations within *V. dahliae* and highlighted changes in both genome and transcriptome associated with lifestyle alterations. Moreover, the study by Rafiei et al. (2018a) focused on analyzing the diversity and structure within subpopulations of *V. dahliae* from various hosts and regions, unveiling distinct patterns among them.

Overall, we have gained insights into the underlying molecular mechanisms behind host pathogen interactions in *V. dahliae* through population genomics. For instance, Wang et al. (2021) explored how different varieties of plants diverge and identified effector genes that play a crucial role in interactions between hosts and pathogens. Another research conducted by Fan et al. (2018) focused on understanding the toxicity of *V. dahliae* compatibility groups (VCGs) in strawberries, highlighting the influence of genetic factors on different compatibility groups (VCGs) virulence. Bautista-Jalón et al. (2021) examined the differences among populations of *V. dahliae* recovered from symptomatic and healthy-looking host plants using microsatellite markers. The findings revealed that populations from symptomatic plants exhibited higher genetic diversity and significant differentiation than those from asymptomatic plants. It is worth noting that different clonal lineages of *V. dahliae* may have developed diverse infection strategies over time, leading to variations in their virulence levels. In Fan et al. (2018) research, genome sequencing has assisted in uncovering differences across various regions of the genome and changes in potential effector genes among distinct isolates belonging to different VCGs within *V. dahliea* populations infecting strawberries, which indicates unique evolutionary approaches employed by this fungus when it comes to infection and affecting their overall virulence. The population structure and the gene flow among different *Verticillium* species significantly impact disease management strategies and contribute to the continuous evolution of these pathogens. According to a study conducted by Baroudy et al. (2019), genetic diversity exists in populations of *V. dahliae* found in olives and potatoes, impacting the presence of effector genes. Another study by Dung et al. (2019) analyzed *V. dahliae* populations isolated from mint and revealed relatively low genetic diversity, suggesting the potential for the evolution of pathogenic genotypes.

In general, population genomics has implications for disease management and agriculture as it influences factors such as pathogenicity, host range and resistance to fungicides. By understanding the structure of populations, we can develop strategies, like breeding for resistance and targeted use of fungicides. However, further research is necessary to assess their impact fully.

### 2.3 Horizontal gene transfer and mobile genetic elements

Horizontal gene transfer (HGT) which involves the exchange of material between organisms, play a crucial role in shaping diversity and aiding adaptation in *Verticillium* species. The movement of mobile genetic elements (MGE) such as plasmids, transposons, and bacteriophages within and between genomes significantly contributes to this process (Klosterman et al., 2011; Shi-Kunne et al., 2019a,b; Depotter et al., 2019; Zhang et al., 2022). These elements facilitate the transfer of genes between bacterial and fungal genomes, actively contributing to pathogen adaptation to environments (Torres et al., 2021; Faino et al., 2016). Many researchers have studied the impact of HGT and MGEs on *Verticillium* species using techniques such as transcriptomics and comparative genomics. For instance, Torres et al. (2021) identified transcripts derived from transposable elements (TEs) that influence gene expression regulation in this plant pathogen. They showed that TE dynamics in *V. dahliae* contribute to genomic variation, correlate with the expression of pathogenicity-related genes, and potentially influence the evolution of adaptive genomic regions.

Furthermore, TEs have been discovered to facilitate the transmission of virulence genes, promoting the development of pathogenic strains (McDonald et al., 2019). These mechanisms play an important role in adaptation and evolution as *Verticillium* species often undergo horizontal gene transfer events (Shi-Kunne et al., 2019a; Klosterman et al., 2011).

Horizontal gene transfer has been observed in *Verticillium* species, where they acquire genes from bacteria. This process enhances the fungus’s pathogenesis-related functions, including cell wall degradation and detoxification (Shi-Kunne et al., 2019a,b). In *Verticillium* genomes, the transfer of genes is also linked to the evolution of virulence. For instance, allelic variations in the *V. dahliae* Ave-1 effector gene and its presence in other fungal pathogens provide evidence of HGTs’ role (Chen et al., 2018). Furthermore, HGT can occur between bacteria and *Verticillium* as well as *Verticillium* with other fungal genera influencing the trajectory of pathogens (Chen et al., 2018). For instance, In *V. longisporum*, a lineage-specific genomic region associated with reduced virulence has been identified. HGT event with TEs as a mechanism is believed to be involved in this region’s evolution (Harting et al., 2021). Moreover, repetitive elements in the *Verticillium* genome, such as centromeres, contribute to diversity and evolution. These repetitive elements facilitate genetic material rearrangement between centromeres and aid adaptation to changing environmental conditions (Seidl et al., 2020).

Aside from HGT and MGE variations, chromosomal rearrangements in *Verticillium*, such as duplications, deletions, or transpositions, are linked to the gain and loss of virulence factors. These alterations impact the fungus’s ability to infect and colonize host plants and contribute to host specificity (de Jonge et al., 2013). The genome size reduction combined with rearrangement events has also been noted in different members of *Verticillium*, potentially influencing their ability to diversify and adapt to various host plants (Shi-Kunne et al., 2018).

Besides the nuclear genome, the mitochondrial genome of *V. longisporum* displays a mosaic structure due to allopolyploidization—a process involving duplication through hybridization between different species known as interspecific hybridization. This specific process is believed to have influenced *V. Longisporum’s* ability from infecting multiple crops to shift toward infecting mainly Brassicaceae plants. The mosaic structure of its genome was primarily caused by rearrangements between parental chromosomes, with gene conversion playing a minor role. The interaction between the mitochondrial and nuclear genomes played a significant role in maintaining genome stability and adapting *V. longisporum* to ecological niche (Depotter et al., 2018).

Understanding the mechanisms behind horizontal gene transfer, mobile genetic elements, and TEs in *Verticillium* genomes is vital for unraveling their diversity, pathogenicity, and ability to infect various hosts and survive in different environmental niches. These studies provide valuable information about the evolution of the genome in *Verticillium*. By gaining these insights, we can potentially develop strategies to combat plant diseases caused by *Verticillium*.

## 3 Small RNA and epigenetic regulation

Small RNA molecules (sRNAs) and epigenetic modifications are important in controlling gene expression and affecting the pathogenicity and virulence of plant pathogens, including *Verticillium* spp. sRNAs regulate gene activity after transcription, while epigenetic modifications involve changes to DNA and histones without altering the DNA sequence itself. Recent studies have underscored the importance of sRNAs and epigenetic modifications in governing genes associated with pathogenicity in *Verticillium* species. For example, Jin et al. (2019) reported that a particular small RNA called VdmilR1 regulates the virulence gene VdPL1 in *V. dahliae* by limiting a protein-coding gene called VdHy1 by an increase in histone H3K9 methylation levels. Furthermore, Kramer et al. (2020) investigated two DNA methyltransferases known as Dim2 and Dnmt5 in *V. dahliae*, finding that Dim2 functions as the enzyme for DNA methylation within its genome. Studies have demonstrated that DNA methylation plays a role in regulating the expression of virulence genes within isolates of *V. dahliae* (Ramírez-Tejero et al., 2020). Additionally, Zhang et al. (2021) revealed that when *V. dahliae* infects cotton plants, they respond by increasing the production of miRNAs (miR166 and miR159) which target two virulence genes specifically. This suggests that host plants export these molecules to silence genes and develop resistance against *Verticillium* infection.

Exploring sRNAs and epigenetic regulation in *Verticillium* can potentially provide approaches for disease control. Small RNAs, including those found in *Verticillium*, influence the interactions between plants and pathogens (Huang et al., 2019). In their work, Qiao et al. (2021) summarize the current understanding of how RNAs mediate plant immunity and influence pathogen virulence. The authors discuss mechanisms through which small RNAs regulate plant responses and pathogen virulence, such as directly controlling gene expression and RNA interfering between domains.

Furthermore, studies have identified small RNAs in *V. nonalfalfae* (Jeseničnik et al., 2022) and small RNA molecules secreted by *V. dahliae* that target the host MIR157d to delay the transition of plant flowers during infection (Zhang et al., 2022). Additionally, sRNAs have been discovered in *V. dahliae* after cotton inoculation, potentially regulating gene expression during infection (Li F. et al., 2022).

These findings highlight the role of epigenetic regulation and sRNA in *Verticillium* virulence and pathogenicity. Further investigation into these mechanisms may lead to strategies for controlling these significant plant pathogenic fungi. For example, future research on sRNAs and epigenetic modifications in *Verticillium* species should focus on identifying specific sRNAs regulating pathogen virulence and host defense genes. Moreover, understanding cross-kingdom sRNA interactions and integrating multi-omics approaches will be crucial for unraveling complex regulatory networks.

## 4 Genomics approaches and complex networks

Genomics approaches and complex networks are crucial for understanding how *V. dahliae’s* complex life cycle evades host defenses and colonizes plant tissues. High throughput methods have provided insights into the mechanisms through which it infects plants (Gabur et al., 2020). Using genomics and transcriptomics approaches, studies on cotton have identified differentially expressed genes and gene coexpression networks in response to *V. dahliae*’s infection (Zhang et al., 2021). In eggplants, transcriptome analysis has revealed increased acid and salicylic acid pathways in resilient plants inoculated with *Verticillium* (Yang et al., 2019). Researchers have employed metabolomics, proteomics, and systems biology to investigate responses to *V. dahliae* (Hu et al., 2019; Wu et al., 2021). Moreover, GhCOMT and NAC genes associated with verticillium wilt resistance were discovered in cotton (Wu et al., 2021; Wang et al., 2016).

Transcriptome analysis of *Olea europaea* roots has unveiled genes activated during the stages of *V. dahliae* infection (Jiménez-Ruiz et al., 2017). Furthermore, Jiménez-Ruiz et al. (2017) identified genes involved in plant defense response and metabolism, including the phenylpropanoid pathway, which may protect plants against pathogens. Cotton plants exhibited increased resistance to *V. dahliae* through host-induced gene silencing techniques (Xu et al., 2018). In wild eggplant species (*Solanum aculeatissimum*), *de novo* sequencing has identified genes associated with defense mechanisms and stress response to *Verticillium* (Zhou et al., 2016). Differential mitogen-activated protein kinase (MAPK) gene expressions were also observed in cotton when inoculated with *V. dahliae* (Meng et al., 2018). Identifying disease-related genes in *Gossypium hirsutum* plays an important role in developing resilient cotton varieties (Zhang et al., 2017). Researchers also discovered that cotton possesses ABC transporter-mediated resistance against *V. dahliae* (Dong et al., 2019). Furthermore, PAMP molecules activated pathogenesis-related genes in cotton plants’ roots (Du et al., 2017). In another study conducted by Su et al. (2018), it became evident that changes in defense-related gene expression in *Arabidopsis* plants during *V. dahliae* infection. Another research effort led to the *de novo* assembly of *Solanum sisymbriifolium* transcriptome, which shed light on defense responses against *Verticillium* infection (Wu et al., 2019).

Genomics applications have offered insights into host-pathogen interactions and potential targets for developing resilient cultivars (Acharya et al., 2020; Zhang et al., 2016). However, challenges remain in comprehending the regulatory mechanisms, underscoring the necessity for continued research. Future investigations should integrate multi-omics approaches to delve into deeper molecular interactions, thereby facilitating the development of resilient crops and pioneering strategies for disease management.

## 5 Functional genomics

Functional genomics provides tools for investigating molecular biological systems, particularly when examining interactions between plants and pathogens. In recent years, this field has experienced huge advancements, which play a vital role in comprehending the mechanisms behind *Verticillium* pathogenesis and host resistance. Cutting-edge approaches such as transcriptomics and proteomics have revealed genes and pathways influencing disease progression and host responses. These groundbreaking findings hold the potential for developing effective environmentally friendly strategies for disease control. In the following subsections, we will delve into functional genomics’ applications in studying *Verticillium* pathogenesis and host resistance.

### 5.1 Comparative transcriptomics

Transcriptomics is important in studying interactions between *Verticillium* fungi and host plants. It provides insights into the pathogenicity mechanisms of these important groups of fungi and how plants defend themselves against them. Researchers have used whole genome sequencing to identify genes that contribute to the virulence of *V. dahliae* and other *Verticillium* species and genes that determine their ability to infect specific hosts. By combining transcriptomics with genomics, Zhang et al. (2020) examined the expression of *V. dahliae*’s genes in cotton root exudates, uncovering pathogenicity and stress response genes. Similar approaches have been employed in studies involving tomato, cotton, and alfalfa (*Medicago sativa*), leading to discoveries and potential targets for disease control (Tan et al., 2015; Zhang et al., 2019; Li J. et al., 2022). A very recent transcriptome study by Zhang et al. (2023) explored the effects of mycosubtilin C17 and Chaetoviridine A on *V. dahliae,* revealing several functional pathways influenced by these antifungal compounds. Furthermore, recent advancements in transcriptomics have allowed for analysis of the dynamics of host-pathogen interactions.

To better understand the disease cycle of *V. dahliae*, researchers have employed different advanced methods such as genetics, transcriptomics and comparative analysis. Sarmiento-Villamil et al. (2020) employed inheritance genetics and transcriptomics to investigate the disease cycle of *V. dahliae*. Through their research, they identified three differentially expressed genes during infection, one of which was VdRGS1—a regulator involved in G protein signaling pathways. Deletion of VdRGS1 had impacts on the pathogen’s development and virulence, highlighting its role in disease progression. It was found that G protein-mediated signaling triggers the production of virulence factors during the biotrophic growth stages of *V. dahliae,* while inhibiting G protein signaling through VdRGS1 is necessary for microsclerotia production later in infection. Targeting the signaling of G proteins could present an approach for controlling diseases. However, additional research is required to develop such methods.

Several transcriptomic analyses have investigated how hosts respond to *V. dahliae* infection. For instance, Jiménez-Ruiz et al. (2019) examined the transcriptome of olive cultivars with varying levels of susceptibility, while Zhu et al. (2021b) analyzed a cotton variety that displayed increased resistance. On the other hand, Li et al. (2023) compared interspecific cotton lines to identify potential genes involved in verticillium wilt resistance. Comparative transcriptomic analyses have also unveiled networks and crucial genes associated with pathogenicity and host defense mechanisms. For example, Tan et al. (2015) delved into the interaction between tomatoes and *V. dahliae*, Guo et al. (2017) compared sunflower genes that respond to *V. dahliae* and Yang et al. (2019) examined transcriptional responses in eggplants. Additionally, Zhao et al. (2020) investigated cross-protection in sunflowers using a weakly aggressive strain of *V. dahliae*, Luo et al. (2019) used transcriptomics to study microsclerotia formation while Wang et al. (2021) analyzed the role of VdBre1 in cotton infection with *V.dahliae*. Moreover, Ramírez-Tejero et al. (2021) focused on olive roots’ basal gene expression for resistance against verticillium wilt and presented insights into understanding mechanisms involved in *V.dahliae* plant interactions.

RNA sequencing (RNA seq) is also used to investigate splicing in interactions between pathogens and their host plants. In a study by Jin et al. (2017), RNA seq was employed to analyze the transcriptomes of *V. dahliae*, revealing that over 50% of its multi-exon genes undergo alternative splicing. Targeting these splicing events might be possible to control infections caused by *V. dahliae* in crops. Similarly, Xiong et al. (2014) investigated microsclerotia development in *V. dahliae* and discovered that intron retention (RI) is involved in more than 95% of alternative splicing events. Jin et al. (2017, 2019) identified differentially expressed genes involved in alternative splicing regulation and virulence, shedding light on the importance of alternative splicing events and their role in fungal virulence. Additional investigation is required to understand the consequences of these events and their significance in the molecular mechanisms underlying microsclerotia development in *V. dahliae*.

In another study, Liu et al. (2022), using transcriptomics, identified VdCf2 as a regulator influencing growth, pathogenicity, and a gene cluster responsible for secondary metabolite production in *V. dahliae*. By controlling the expression of this gene cluster, VdCf2 impacts virulence and provides targets for antifungal drugs. Additionally, Liang et al. (2021) revealed the role of Nbnrp1 in defense responses triggered by the PevD1 effector from *V. dahliae* in *Nicotiana benthamiana* as a model organism. Nbnrp1 regulates sesquiterpenoid phytoalexin biosynthesis, inhibiting the growth of *V. dahliae* highlighting the importance of effector-triggered defense responses in plant immunity. Through examining the effector gene expression, Santhanam and Thomma (2013) investigated the role of Sge1. They found that it positively regulates some effector genes while repressing others—demonstrating the complex nature of effector gene regulation in *V. dahliae*.

Furthermore Zhang et al. (2020) examined how *V. dahliae* responds to cotton root exudates, establishing connections between this response and secondary metabolism as virulence. In another research, Tang et al. (2021) investigated the transcriptional variations in *V. dahliae* under different sources of inorganic nitrogen, whereas Wang et al. (2021) delved into understanding the importance of VdBre1 during cotton infection. In recent transcriptomic research, an unusual protein called VdTrx1 was identified, which is secreted and acts as a factor contributing to virulence as its deletion led to reduced pathogenicity (Tian et al., 2023). Another study by Zhang et al. (2020) shed light on the mechanisms underlying the interaction between *V. dahliae* and its host plant, providing insights into disease control strategies. These studies highlight the importance of transcriptome analysis in understanding *Verticillium* pathogenesis and host resistance, thus aiding in the development of effective control measures.

### 5.2 Proteomics

In the genomics era, proteomics has emerged as a crucial and powerful tool for understanding the complexities of *Verticillium* pathogenesis. Proteomic analysis offers a view of the events that shape the dynamics between hosts and pathogens by investigating protein expression, interactions, modifications, and functions during infection. Hu et al. (2019) conducted proteome and metabolome analyses to explore the interaction between tomatoes and *V. dahliae,* uncovering changes in defense-related proteins. In a study, Zhang et al. (2019) utilized phosphor proteomics to examine how cotton roots respond to *V. dahliae,* identifying phosphorylated proteins involved in defense and signaling pathways. Besides enhancing our understanding of host responses, phosphor proteomics provides information on targets for modifying these pathways to improve disease resistance.

Exo proteomics has also played an essential role in identifying pathogenicity factors within *V. dahliae*. This method offers insights into the proteins that contribute to the pathogen’s virulence ability. Such knowledge is crucial for developing targeted interventions that disrupt these pathogenicity factors and pave the way for strategies for preventing infections. Recent research conducted by Tian et al. (2023) identified Thoredox VdTrx1 as a factor that decreases the host’s immunity, thus contributing to the virulence of *Verticillium*. The discovery of Thoredox VdTrx1 in *Verticillium* as a virulence factor adds to the puzzle shedding light on the intricate molecular strategies *Verticillium* uses to establish infections. This protein’s discovery has been opening avenues for further research and potential interventions aimed at disease control.

Effector proteins also play a crucial role in *Verticillium* pathogenesis, as explained by de Sain and Rep (2015). These proteins unpredictably alter host pathways and cell structures. For example, the discovery of XFORCE1 revealed its targeting of plant mRNA turnover and its ability to induce changes in cell identity (Subieta, 2023). Such examples highlight the various ways effectors undermine host defenses. Additionally, an effector found in *V. dahliae* that influences tomato pathogenicity through its interaction with auxin response factors provides evidence of the interplay between this microbe and its host (Li F. et al., 2022).

In conclusion, the studies mentioned above collectively emphasize proteomics’ role in understanding the molecular mechanisms involved in *Verticillium* pathogenesis. The intricate concept of the information derived from studies enhances our understanding of *Verticillium* host interactions and provides a new opportunity for developing targeted approaches to control diseases. As we delve deeper into the proteomics realm, it becomes necessary to conduct further research to unravel the complex network of molecular events, identify new effectors, and explore potential targets for mitigating the impact of verticillium wilt in agriculture.

## 6 Advanced genomic techniques in *Verticillium* studies

### 6.1 Genome-wide association studies

The advent of high throughput sequencing techniques as well as the availability of reference genomes have presented the new era, in unraveling the genetic foundations of plant disease resistance through genome wide association studies (GWAS) (Song et al., 2020). In the case of Verticillium, GWAS has emerged as a tool for identifying genes and pathways associated with resistance across different plant species. For instance, Cotton was the subject of a GWAS study where the scientists uncovered genes that are associated with Verticillium resistance (Li et al., 2017). Following studies focused on *Gossypium arboreum* (Gong et al., 2018) and *G. barbadense* (Li et al., 2018) expanded upon this idea improving our understanding of factors related to resistance in cotton cultivars. Remarkably, an inclusive GWAS analysis conducted to identify quantitative trait loci (QTL) associated with resistance against both Verticillium and fusarium race 4 in upland cotton (Abdelraheem et al., 2020). The discovery of QTLs holds promise for developing cotton cultivars with enhanced resistance against Verticillium through marker assisted breeding.

The broad application of GWAS enables researchers to identify factors that confer resistance in plant species offering a more extensive perspective on the mechanisms underlying Verticillium resistance. For example, GWAS was also employed in autotetraploid alfalfa (*Medicago sativa* L.) to discover markers, for verticillium wilt resistance (Li et al., 2018). This represents a big step, in the development of strategies to breed alfalfa cultivars with increased resistance to Verticillium. Furthermore, GWAS, QTL seq and transcriptome sequencing techniques were employed to identify candidate genes and develop markers for breeding Verticillium varieties (Zhao et al., 2021). Their work showcased the integration of different cutting-edge approaches for a more comprehensive understanding of Verticillium-host interactions. In a recent study, Fartash et al. (2023) pinpointed loci associated with quantitative resistance in *Medicago truncatula* to *V. Alfalfae* thereby contributing to enhanced resistance.

Apart from identifying genetic factors GWAS has also played a vital role in unraveling the intricate molecular aspects of Verticillium pathogenesis. For example, Zhou et al. (2023) investigated heat shock protein genes in cotton, revealed their response to biotic stress and infection caused by *V. dahliae*. This highlights how GWAS can uncover not just resistance related genes but also provide insights into the mechanisms involved in host pathogen interactions.

In general, GWAS remains a strong methodology for discovery of the genetic factors associated with Verticillium wilt resistance. The studies mentioned above provide a path, for the development of resilient plant cultivars and the enhancement of strategies to control diseases caused by different Verticillium species. As we continue to advance our knowledge about the factors behind plant resistance, it is likely that GWAS will play a role in determining the future of crop breeding and contributing to worldwide initiatives aimed at minimizing the negative effects of Verticillium wilt, on agricultural productivity.

### 6.2 Genetic transformation of *Verticillium dahlia*

Genetic modification is a great technique to better understand the factors contributing to the pathogenicity and virulence of plant pathogens. The ability to introduce foreign DNA into *V. dahliae’s* genome has greatly aided in studying the genes for its virulence and pathogenicity (Rehman et al., 2016). Comparative studies on the genome of *V. dahliae* have revealed diversity and rearrangement within its chromosomes highlighting the importance of comprehending its genetic composition (de Jonge et al., 2013). Moreover, as explained earlier, by examining assemblies of *V. dahliae* researchers have identified genomic regions that are highly flexible and enriched with TEs, duplicated genes, as well as genes involved in signaling and transcriptional regulation which provide valuable insights for potential genetic modification studies (Klosterman et al., 2011). The emphasis on resistance to combat Verticillium wilt diseases underscores the significance of using genetic modification techniques to unravel mechanisms related to resistance and pathogenicity (Santhanam et al., 2017). It is widely acknowledged that genetic transformation serves as a platform for exploring gene function in *V. dahliae* enabling us to uncover information about its virulence and pathogenicity at a genetic level (Rehman et al., 2016). Therefore, genetic transformation plays a role in unraveling the underlying genetics behind pathogenicity, host interactions and identifying strategies, for disease control. In this section we will explore research on Verticillium focusing on significant methods of transformation that have been utilized.

#### 6.2.1 Protoplast-based transformation method

Protoplast based transformation is a great method for manipulating the genetics of Verticillium species. This technique has greatly contributed to the exploration of different aspects of Verticillium biology, such as pathogenicity and responses to stress. For instance, researchers have identified two LysM effectors, VdLYS1 and VdLYS2 which play an important role in enhancing fungal virulence in cotton and tomato plants. However, the impact on virulence was found to be negligible for VdLYS3 (Kombrink et al., 2017). Studies on components of the mitogen activated protein kinase (MAPK) pathway, namely VdSsk2 and VdSte11 have revealed their roles in pathogenicity, microsclerotia formation as well as adaptation to biotic stress (Yu et al., 2019). Similarly, transcription factors such as VdMcm1—a MADS box transcription factor—and STT3—an oligosaccharyltransferase subunit—have been discovered to play roles in regulating fungal development and pathogenicity (Xiong et al., 2016; Su et al., 2018). Furthermore, our understanding of iron metabolism in *V. dahliae* has been enriched through the identification of FreB as a regulator of iron uptake and pathogenicity (Rehman et al., 2018). In a study conducted on *Nicotiana benthamiana* plants, Su et al. (2018) employed mCherry tagged *V. dahliae* for real time observation to gain insights, into the behavior of this important plant pathogen during infection process. Similarly, a study conducted by Xiong et al. (2018) demonstrated how knocking out of VdKu80 gene enhances gene replacement contributing to our understanding of virulence mechanisms. Manipulating genes in an effective tool to functionally identify genetic targets for disease control. Furthermore, various other studies have examined the roles of genes in *V. dahliae*’s pathogenicity and virulence. For instance, Xiao et al. (2023) discovered that GhWRKY41 plays a role in cotton defense against *V. dahliae* through phenylpropanoid metabolism. Understanding plant defense responses helps us enhance resistance against this pathogen. Moreover, the role of the VdASP F2 interacting protein in microsclerotia formation has been identified in *V. dahlia* (Guo et al., 2022). These studies highlight the significance of genomics in identifying genes and pathways that drive pathogenicity and provide valuable insights for controlling *V. dahliae* related plant diseases. Genomics has also enabled us to study how *V. dahliae* responds to stressors. Fang et al. (2019) investigated the role of VdCmr1 in protecting the pathogen against temperature and UV radiation— environmental stressors that impact fungal survival. Furthermore, the importance of the Ada1 subunit within the Spt-Ada-Gcn5 acetyltransferase complex for conidia and microsclerotia production—a process for both pathogen survival and virulence, has been comprehensively studied (Geng et al., 2022). These genomic studies provide insights, into epigenetic mechanisms that regulate gene expression in fungi.

#### 6.2.2 Agrobacterium-mediated transformation method

The technique known as Agrobacterium mediated transformation has been successfully utilized for *V. dahliae* providing insights, into genes related to pathogenicity. For instance, a protein called VdSCP7, which is specific to Verticillium has been discovered to alter the response in host plants (Zhang et al., 2017). Using Agrobacterium mediated transformation, researchers have also identified VdCYP1, an enzyme belonging to the P450 family as a regulator of oxidative stress and virulence factor in *V. dahliae* (Zhang et al., 2016). Additionally, transcription factors like VdFTF1 have been found to control the expression of secreted virulence factors that play a role in pathogenicity of Verticillium on cotton plants (Zhang et al., 2018). The exploration of genes involved in production of melanin and microsclerotia formation, such as Vayg1, VdPKS1, and VDAG_07742 has significantly expanded our knowledge about the survival strategies employed by *V. dahliae* (Fan et al., 2018; Zhang et al., 2017; Wang et al., 2023). Moreover, using Agrobacterium mediated transformation approach, researchers have determined that a regulator called VdCf2 plays a role in governing growth patterns and pathogenicity while influencing the expression of secondary metabolic genes (Liu et al., 2022). This finding underscores the significance of transcription factors, in pathogenesis and highlights how functional genomics can aid in identifying regulators of virulence within *V. dahliae*.

It is evident that *V. dahliae* has evolved mechanisms to regulate both virulence traits and growth-related characteristics. For example, researchers discovered that the G protein’s *β* subunit plays a role, in regulating virulence of this important plant pathogenic fungus (Tzima et al., 2012). When the gene for the Gβ subunit was deleted, it resulted in virulence suppression, changes in conidiation (spore formation) and impaired fungal growth. This highlights how important this signaling pathway is for complete infection. Moreover, VdMsb, was also found to be vital for controlling virulence and microsclerotia production (Tian et al., 2014). Deleting of VdMsb led to reduced virulence and microsclerotia production underscoring its role in the fungus’s ability to cause disease and survive.

Recent research by Kramer et al. (2021) investigated DNA methylation event in Verticillium. They observed differences in patterns between wild type and mutant strains suggesting that alternative mechanisms could regulate gene expression and affect virulence in this fungus. However, the impact of DNA methylation on the growth, development, and virulence of *V. dahliae* is not yet fully understood. Furthermore, Höfer et al. (2021) explored the Vel1 protein, found it to be indispensable for establishing *V. dahliae* on plant roots as well as for conidia formation. Vel1 also controls genes involved in producing secondary metabolite metabolites with antifungal properties.

Through transformation studies and functional genomics research scientists have identified many pathogenicity related genes, transcription factors and proteins involved in manipulating plant immunity during verticillium wilt infection. These findings offer targets that could be explored to combat this disease. However more studies are required to gain a better understanding of the dynamics, between the pathogen and the host as well as to devise efficient and long-lasting management methods.

#### 6.2.3 CRISPR-Cas9 transformation method

CRISPR Cas9 technology has brought about a huge transformation in the field of molecular biology. It has enabled targeted editing of genomes, facilitated the discovery of genes that play a role in plant pathogen interactions. CRISPR Cas9 gene editing has successfully enhanced canola resistance to *V. longisporum* by disrupting susceptibility genes within the plant genome thereby reducing the virulence of the pathogen (Pröbsting et al., 2020). Moreover, the combination of CRISPR/Cas12a technology, a new RNA guided Cas enzyme-based method that has been recently introduced as an alternative genome editing tool, with recombinant polymerase amplification (RPA) has shown potential in quickly and accurately detecting *V. dahliae* in samples opening possibilities for early identification of pathogens and effective treatment strategies (Wang et al., 2022). These studies highlight how CRISPR based genome editing can provide insights into understanding the mechanisms behind plant pathogen interactions and developing crop varieties resistant to diseases like verticillium wilt. Through targeted gene editing and enhancing resistance, CRISPR Cas9 technology holds promise for combating devastating diseases such as Verticillium wilt.

In summary, genomics and functional genomics approaches have completely transformed our understanding of Verticillium pathogenesis and host resistance. Techniques such as protoplast-based methods Agrobacterium mediated approaches and CRISPR Cas9 transformation have played a role, in this revolution. These tools have allowed researchers to identify genes and pathways that influence fungal pathogenicity, virulence as well as stress responses. Additionally, genomics tools have provided insights into unraveling the molecular mechanisms underlying host pathogen interactions. This newfound knowledge has opened up possibilities, for creating strategies to control diseases and produce crop varieties that are resistant to verticillium wilt and other similar fungal pathogens. It is vital to conduct more research and develop new tools in order to combat the destructive effects of plant diseases and enhance crop protection.

## 7 Genomics in *Verticillium* control: strategies and management

Genomic research has played a crucial role in advancing our understanding of innovative strategies for controlling Verticillium related diseases. One important approach involves using studies to identify candidate resistance genes, which can then be used in breeding programs to develop more resilient plant varieties. Exciting discoveries of Verticillium resistance genes have been made in plant species like cotton, tomato, and strawberry (Li et al., 2021; Chavarro-Carrero et al., 2021) which deepen our knowledge and potential for breeding and genetic engineering.

Another effective management strategy is the use of biopesticides to suppress Verticillium populations. Genomic research has identified strains, such as *Bacillus velezensis* AL7 and Bacillus sp. Strain BS Z15, that show remarkable potential for biocontrol against verticillium wilt in cotton (Liu et al., 2021; Chen et al., 2021). Understanding the basis of their biocontrol activities allows us to optimize their use in the field and develop potent strategies.

Furthermore, genomics has provided insights into the virulence factors and signaling pathways in Verticillium fungi. This knowledge opens up possibilities for developing fungicides by targeting these genetic factors. For instance, Wang et al. (2021) demonstrated how functional genomics and comparative analysis can help identify differences within Verticillium fungi potentially aiding the design of fungicides that are specific to particular races. In a recent study, Liu et al. (2023) made a groundbreaking discovery by identifying VdEPG1, a virulence factor in *V. dahliae*. They found that it interacts with GhOPR9 a gene associated with the jasmonic acid pathway. By targeting this interaction there is potential for the development of fungicides that can inhibit virulence. In another study Maurus et al. (2023) explored the role of Vta3, a protein produced by Verticillium fungi. This protein shows promise as a target to develop antifungal agents. Additionally, Zhang et al. Discovered VdHP1, a hydrophobic protein that suggests a strategy for developing fungicides with reduced impact by preventing early infection. Both VdEPG1 and VdTrx1 proteins play roles as virulence factors (Liu et al., 2023; Tian et al., 2023). This finding opens up possibilities for developing agents that can specifically disrupt the functions of these proteins and ultimately reduce disease severity.

Genomics has also played a crucial role in creating tools for rapid and accurate detection of Verticillium species. Advanced techniques such, as polymerase chain reaction (qPCR) (Lee et al., 2022), Inter Simple Sequence Repeats (ISSR) analysis (Dadd-Daigle et al., 2022) and digital PCR (dPCR) (Wang et al., 2022) have been utilized to detect and quantify Verticillium pathogens. Furthermore, the use of species SSR markers has proven to be effective, in accurately identifying different Verticillium species (Jeseničnik et al., 2023). This enables intervention and targeted measures for disease control.

The application of genomics in studying the population structures and gene flow of Verticillium species also plays a role in implementing focused and efficient control methods. By monitoring the emergence and spread of new Verticillium strains with varying virulence or resistance characteristics we can take steps in managing these issues. Integrating genomics into Verticillium control not only expands our understanding of this pathogen, but also allows us to develop innovative management strategies thereby enhancing agricultural yields. However, to fully harness this potential, continuous research and development are necessary to improve and optimize the application of genomic tools in the field.

## 8 Conclusion

The study of verticillium wilt disease, particularly caused by *V. dahliae*, has greatly benefited from the integration of genomics and functional genomics’ approaches. These advanced techniques have provided deep insights into the molecular mechanisms underlying pathogen virulence and host plant defense. Through multi-omics approaches, including transcriptomics, proteomics and genomics, scientists have identified key genes, pathways and factors contributing to Verticillium pathogenesis as well as host responses. Genome wide association studies (GWAS) have also identified resistance genes in various plant species, opening up possibilities for developing resistant crop varieties through breeding or genetic engineering. Furthermore, genomics research has enhanced our understanding of Verticillium species’ diversity and population structure, thereby clearing the path for further development of targeted control measures. These efforts include the identification of effective biocontrol agents and improved diagnostics, enabling early intervention and precise disease management. The advancements in gene editing technologies such as CRISPR Cas9 have further empowered researchers to manipulate Verticillium genomes, facilitating the identification of genes critical for fungal survival and virulence.

The significance of genomics in advancing Verticillium research cannot be overstated. By unraveling the complex genetic underpinnings of this pathogen, genomics has opened new avenues for innovative disease management strategies that promise to enhance agricultural productivity. From the development of resilient crop varieties to the precise application of biocontrol agents, the insights gained from genomics are instrumental in building more sustainable and effective approaches to managing Verticillium wilt.

In summary, the incorporation of genomics into the study of Verticillium wilt has significantly advanced our understanding of pathogenic Verticillium species and their interactions with host plants. These insights have paved the way for innovative disease management strategies, including the development of resistant crop varieties, targeted fungicides, and biocontrol agents. Ongoing genomics research will continue to play a crucial role in combating Verticillium wilt and improving agricultural productivity.

## 9 Future perspectives

The field of genomics of Verticillium is constantly growing. There are new tools and technologies that hold great potential for enhancing our understanding of this important fungal pathogen. One area of research that is particularly promising is single cell genomics, which allows for an exploration of the genetic diversity and evolutionary patterns within Verticillium populations. This approach has the potential to uncover genes and pathways involved in disease development leading to therapeutic strategies. In addition to single cell sequencing, machine learning and artificial intelligence offer prospects for advancing Verticillium research. These cutting-edge technologies can analyze huge amounts of data providing insights into the molecular mechanisms underlying Verticillium behavior. By predicting virulence in strains and developing targeted control strategies researchers can make better progress in combating this pathogen. Another emerging technology known as read sequencing has also proven to be highly effective in assembling high-quality full-length genomes. This approach is especially advantageous when dealing with fungal genomes like those found in Verticillium species. Read sequencing has already demonstrated success in plant pathogenic fungi and is expected to become an essential tool in studying Verticillium genomes.

In general, the future of Verticillium research looks bright as tools and technologies continue to expand our understanding of its biology and evolution. By employing these advancements, researchers can develop efficient sustainable strategies for controlling and managing Verticillium infections thus safeguarding crop production and ensuring food security for future generations.
